# Adolescent psychopathology and psychological wellbeing: a network analysis approach

**DOI:** 10.1186/s12888-021-03331-x

**Published:** 2021-07-03

**Authors:** Stephanie Campbell, Tom L. Osborn

**Affiliations:** Shamiri Institute, Nairobi, Kenya

**Keywords:** Adolescents, Africa, Network analysis, Psychopathology, Mental health, Depression, Anxiety, Well-being, Happiness, Social support

## Abstract

**Background:**

The extent to which psychological wellbeing may play a preventive and therapeutic role in the development and maintenance of adolescent emotional disorders depends, in part, on the nature of the overlap between these two constructs. We estimated network analysis to examine the relationship between adolescent psychopathology (measured by depression and anxiety symptoms) and psychological wellbeing (measured by happiness, optimism, social support, perceived control, and gratitude).

**Methods:**

This was a cross-sectional study with a large community sample of Kenyan adolescents (*N* = 2192, aged 13–18). Network analyses were conducted to examine the topology, stability, centrality, and bridge nodes of a network of psychopathology and psychological wellbeing measures.

**Results:**

Two distinct community clusters emerged, one for psychopathology nodes and another for wellbeing nodes, suggesting that these are two distinct but connected concepts. Central and bridge nodes of the wellbeing and psychopathology network were identified. The most central nodes in the network were *family provides emotional help and support* and *self-blame;* the strongest negative edges between psychopathology and psychological wellbeing were *depressed mood—I love life* and *irritability—I am a joyful person;* the main bridge nodes were *family helps me* and *I can talk to family about problems.*

**Conclusions:**

Our findings expand understanding of the relationship between psychopathology and wellbeing in an understudied population and are suggestive of how psychological wellbeing can inform psychopathological treatment and preventive efforts in low-income regions such as those in Sub Saharan Africa.

**Supplementary Information:**

The online version contains supplementary material available at 10.1186/s12888-021-03331-x.

## Background

Adolescent mood and anxiety disorders affect a significant portion of the global adolescent population, accounting for 45% of the global disease burden on youths aged 15–19 [1]. Even below a clinical threshold, depression and anxiety are associated with many negative physical, social, behavioral, and health outcomes, which can last a lifetime [2, 3], such as increased risks in health, behavior, and education [4]. Nearly half of all behavioral and emotional disorders have an initial onset during adolescence [5], increasing the risk of these disorders in adulthood by 2-to-3 times [6]. Therefore, this transitional phase of adolescence is a critical time to prevent and treat emotional disorders like depression and anxiety.

One approach to combat youth mental health disorders might lie in the promotion of positive aspects of psychological functioning, such as psychological wellbeing, which may play a preventative and therapeutic role in the development of emotional disorders [7, 8]. Broadly defined, psychological wellbeing refers to an individual’s condition of experiencing their lives positively [9]. It can include the positive feelings of satisfaction, accomplishment, and fulfillment in diverse life domains as well as the lack of negative aspects like mental illness and loneliness [10]. Given its multiplicity, psychological wellbeing is measured by many models, including PERMA (Positive Emotion, Engagement, Relationships, Meaning and Accomplishment; [11]), subjective wellbeing (SWB; [12]), and community quality of life (QoL; [13]). Gratitude, social support, self-perception, and happiness have also been used to measure psychological wellbeing [14, 15].

The extent to which psychological wellbeing plays a preventative or therapeutic role in adolescent psychopathology depends partly on the nature of the overlap between the two [7]. Many studies have shown that psychological wellbeing and psychopathology are strongly and negatively associated [16, 17]. Indeed, the strength of this association—generally, correlations of *r* = − 0.40 to *r* = − 0.55—suggests that these two constructs may be two distinct but overlapping dimensions of functioning [7]. Observers have described this relationship through a binary continuum model, as well as through a dual-factor model. In the binary continuum model, psychological wellbeing and psychopathology are on the extreme opposite ends of a wellbeing band [18]. In the dual-factor model, the two constructs are distinct but related [8, 19]. One study assessed Australian adolescents (*N* = 345) for psychological wellbeing (measured by happiness, life satisfaction, and positive affect scales) and psychopathology (measured by depression, anxiety, and negative affect scales), which revealed two factors for psychological wellbeing and youth psychopathology, respectively [20]. Another study used the dual-factor model along with self-report scores to classify Canadian youths (*N* = 407) into four categories: (1) high psychological wellbeing and low psychopathy, (2) low psychological wellbeing and high psychopathy, (3) low psychological wellbeing and low psychopathy, and (4) high psychological wellbeing and high psychopathy [8]. Another study with American adolescents (*N* = 349) found that by using the cutoff norms for the Child Behavior Checklist and corresponding psychological wellbeing scores, adolescents could also be classified into one of the four groups mentioned above, providing further evidence that psychological wellbeing and adolescent psychopathology may be distinct but interrelated [19].

Beyond the evidence from many cross-sectional studies, one recent Dutch study investigated the extent to which the association between psychological wellbeing and psychopathology is a function of correlated genetic and/or correlated environmental factors [7]. In the study, psychological wellbeing was assessed (measured by subjective happiness, quality of life, and satisfaction with life) for a large population-based cohort of adolescent twins and their non-twin siblings (*N* = 9136 and 1474, respectively). Psychopathology was also assessed (measured by all syndrome and broad band based scales of the Achenbach System of Empirically Based Assessments (ASEBA) Youth Self Report Scale). The study found significant negative associations between psychological wellbeing and psychopathology, which were primarily explained by genetic correlations [7]. This finding––that a genetic liability to lower levels of psychological wellbeing may be suggestive of genetic liability to higher levels of psychopathology––supports the use of psychological wellbeing measures to screen for adolescent psychopathology before the presence of clear signs of psychopathology [7].

The use of psychological wellbeing indices to combat adolescent psychopathology may be of particular importance for adolescent populations in Sub Saharan Africa (SSA). Not only are there high prevalence rates of adolescent depression and anxiety symptoms in SSA countries [21], there is currently a dearth of research with this population, limiting the knowledge of rates, comorbidity, correlates, predictors, protective factors, and treatment options in this region [22]. In SSA, treatment options for psychopathology are limited [2, 21], government spending on mental healthcare is minimal [21], and societal stigma against mental illnesses dissuades many from seeking help [23]. Indeed, research that espouses the association between psychological wellbeing and adolescent psychopathology with SSA populations may be of public policy utility in this region. For example, the societal stigma around mental health limits help-seeking amongst Kenyan adolescents, many of whom do not want to be diagnosed with mental disorders, much less seek treatment for it [23]. If we could use psychological wellbeing indices (like gratitude and happiness) to inform the public health efforts on the screening, prevention, and treatments for youth mental disorders, then these efforts could be done in a potentially non-stigmatizing manner.

One way of expanding our knowledge of the relationship between psychological wellbeing and adolescent psychopathology might lie in the use of network analysis—a novel conceptual model in which a psychological construct is conceptualized as the interplay of traits or symptoms that influence each other [24]. In a network structure, a psychological construct (e.g., a symptom or a trait) is represented by a node, and the relationship between each pair of constructs is depicted by an edge between the corresponding nodes. Networks allow for the identification of the central symptoms or traits of a psychological construct, which are likely to activate the entire network and might be sites for direct targeting in prevention and treatment [25]. The network framework has been used to circumvent some of the theoretical and psychometric limitations of traditional models [26], such as the classification of the symptoms into discrete mental disorders by the DSM [27]. Classification models are problematic since mental disorders share a broad spectrum of overlapping symptoms [24] and since specific disorders may have a wide range of symptoms with different treatments [28]. As network analysis conceptualizes psychiatric disorders as systems the emerge from symptoms interactions and not an underlying disease entity, they embrace and account for the comorbidity and heterogeneity of emotional disorders [29].

In addition to identifying and targeting the central symptoms of an emotional disorder for reduction and the central traits of psychological wellbeing for enhancement, network analysis can help shed light on the connectivity between psychological wellbeing and adolescent psychopathology. Specifically, we can identify an individual psychological wellbeing trait that is highly connected to a particular emotional disorder symptom and quantify the nature of that connectivity. Overall, network analysis can allow us to study the structure of psychopathology and psychological wellbeing, jointly.

In the present study, we used network analysis to analyze the structure of psychological wellbeing indicators and symptoms of depression and anxiety in a large community sample of adolescents in Kenya. Happiness, gratitude, optimism, perceived control, and social support measures were used to assess psychological wellbeing, while depression and anxiety symptoms were used to assess adolescent psychopathology. The aims of our study were to investigate: (1) the structures and clusters that the indicators of psychological wellbeing and the symptoms of depression and anxiety form in a network––i.e., whether psychological wellbeing items form a distinct cluster or overlap with the symptoms of psychopathology, (2) the central nodes (i.e., symptoms or traits) in a network of psychological wellbeing and youth psychopathology, and (3) the important “bridge” nodes that connect the community cluster of psychological wellbeing and the community cluster of adolescent psychopathology. The present study is, to the best of our knowledge, the first of its kind to investigate psychological wellbeing and youth depression and anxiety symptoms in a community sample of SSA adolescents.

## Methods

### Participants and procedures

Participants were 2192 Kenyan adolescents recruited from four secondary schools in Nairobi and Kiambu Counties, as part of a large-scale clinical trial (called *Shamiri* [30, 31];). The trial was registered in the Pan African Clinical Trials Registry (PACTR201906525818462). Participants provided self-report data on depression, anxiety, psychological wellbeing measures, and other health-related and socio-demographic variables by completing a baseline questionnaire battery in their classrooms. There was a slight female majority *(N* = 1246; 58.3%), and the mean age was 15.21 (SD = 1.14). See Table 1 for full demographic information and descriptive statistics. Using a clinical cutoff of 10—per clinical guidelines from North American samples [34, 35] that have been used previously with Kenyan youths [36, 37], some 28.56% of participants (*N* = 626) met the criteria for moderate-to-severe depression and 26.55% (*N* = 582) met that of moderate-to-severe anxiety. All study procedures were approved by the Maseno University Ethics Review Committee (MUERC, No. MUERC/727/19) before the start of data collection. All adolescents were eligible to participate if they consented and were enrolled in the participating schools. Parental consent and written informed consent and assent were obtained for all adolescents per research ethics procedures at MUERC. Data used for the present study is stored in the Open Science Framework repository and is publicly available (DOI: 10.17605/OSF.IO/8M5D9).

**Table 1:** Participant demographic information

Characteristic	N (%)	M (SD)	Range
Age		15.21 (1.14)	14–20
Gender			
Female	1246 (56.80)		
Male	890 (40.60)		
School type			
National	1715 (78.2)		
Rural (sub-county)	476 (21.7)		

More information regarding school type classification in Kenya is available elsewhere [32, 33]

### Measures

#### Adolescent psychopathology measures

Depressive symptoms were assessed using the 8-item version of the *Patient Health Questionnaire* (PHQ-9; [34, 38]), a self-report questionnaire used to assess the severity of depressive symptoms. PHQ-8 and PHQ-9 scores are highly correlated, and the same cutoffs can be used to assess depression severity [38]. A previous study has documented that the PHQ-8 demonstrated adequate psychometric properties for the PHQ-8 with Kenyan adolescents [32, 39] and adults [40, 41]. Anxiety symptoms were assessed using a 7-item self-report questionnaire, the *Generalized Anxiety Disorder Screener* (GAD-7; [35]). Like with the PHQ-8, a previous has documented adequate psychometric properties with Kenyan youths [32].

#### Psychological wellbeing measures

Happiness and optimism were assessed using two sub-scales of the *EPOCH Measure of Adolescent of Well-Being* (EPOCH; [42]): optimism and happiness. Social support was self-reported using the Family, Friends, and Significant Other sub-scales of the *Multidimensional Scale of Perceived Social Support* (MSSS; [43]). Perceived academic control measures adolescent perception of their competency and regulation over that competency and was assessed using the academic sub-scale of the *Perceived Control Scale* (PSC; [44]). Gratitude was measured using the 6-item *Gratitude Questionnaire* (GQ-6; [45]). Gratitude is strongly related to well-being and mental health, a link that has been suggested to be unique and causal [46]. We also collected sociodemographic information such as age, gender, form, tribe, county, economic status, parental education, and family members.

#### Statistical analysis

We performed all our analysis in *R*. First, we investigated the presence of redundant nodes, or overlapping symptoms, by checking pairs of nodes for high correlation (r > .5) and similar correlation patterns with all the other nodes via the *networktools* package in *R* [47]. Second, we estimated the network models for the data and validated their accuracy and stability using the *bootnet* package in *R* [48]. Third, we plotted the network structures and computed the centrality measures using the *qgraph* package in *R* [49]. Fourth and lastly, we investigated bridge symptoms, the main symptoms that connect clusters of symptoms, with the *networktools* package in *R* [47].

To deal with missingness, we conducted multiple imputations using Fully Conditional Specification (FCS) for missing item-level data that was implemented using the multivariate imputation by chain equation (mice) algorithm in *R* [50, 51] under the assumption that data were missing at random. Specifically, we used the predictive mean matching approach to impute missing-item level data fifty times; estimates were then pooled to get one overall set of parameter estimates. The *R* code for our statistical analysis is publicly available (DOI: 10.17605/OSF.IO/8M5D9).

#### Node selection

The lack of variance in variables may lead to the misinterpretation of network structure. If two nodes represent the same construct, there will be redundancy in the network, which can be problematic for interpretability [25]. Thus, redundant pairs should not be included together in the network. In contrast, if two nodes represent independent constructs, we would expect their correlation patterns with other nodes to vary. We tested for the problematic presence of multiple symptoms representing the same underlying construct using the *goldbricker* procedure [47], which checks each pair of nodes for two indicators of redundancy: a high correlation between them (*r* > .5) and a proportion of significantly different correlations with all other nodes under a certain threshold (we used a 20% threshold).

#### Network estimation and accuracy

In psychopathology, a network consists of symptoms and the psychometric associations between them. These associations are not explicitly present in a dataset, but they can be estimated by computing partial correlations between the symptoms, controlling for all other symptom connections [48]. We used *partial.r,* which finds the residuals of various correlations and then correlates these residuals to partial the effect of variables like gender, age, and school type. Next, we estimated a Gaussian graphical model (GGM) to estimate regularized partial correlation networks for psychological wellbeing symptoms and psychiatric symptoms using the *bootnet* package in *R* [29, 48]. We regularized the GGM with the graphical Least Absolute Shrinkage and Selection Operator (LASSO) method to find the best-fitting by penalizing, or shrinking, small edge values estimated in the network. LASSO also helps address the multiple testing problem by controlling false-positive errors. (LASSO regularization techniques are commonly performed in this type of network analysis [52, 53].) The best-fitting model was found with the *EBICglasso* procedure, which selects the optimal degree of shrinkage according to an Extended Bayesian Information Criterion (EBIC) and hyperparameter set to 0.5 [54]. To plot the networks, we used the *qgraph* package in *R*.

#### Node centrality

A highly central node is a node that has particular structural importance in the network based on the strength of its connection to other nodes. The centrality of a node can be used to infer its influence, or structural importance, in the network. There are many indices used to estimate centrality: *betweenness*—how a node influences the average path between other pairs of nodes, *closeness*—how a node is indirectly connected to the other nodes, *strength*—how a node is directly connected to the other nodes, and *expected influence (EI)*—how a node is connected to the sum of all edge weights [55]. As partial correlations were used to estimate the networks, it was not fitting to use the *closeness* or *betweenness* indices. Instead, we relied on the *strength* index to estimate the symptoms that should be most directly targeted in treatment. The *qgraph* package in *R* was used [48].

#### Network accuracy and stability

Bootstrapping was used to determine the accuracy and stability of the networks [48]. First, we tested the accuracy of each network using nonparametric bootstrapping, a process that repeatedly resamples subsets of the data to calculate a confidence interval (CI) as the range of bootstrapped values from different sampling levels. We first used 15,000 bootstraps––or 15,000 repetitions of estimating the model with sampled data and calculating the 95% confidence intervals (CIs)––to assess the accuracy of edge-weights. A large CI indicates that it may be difficult to interpret the edge weight, while a small CI can be interpreted as a precise estimation. Additionally, we enriched the assessment of node importance within the network by using the predictability index, which measures the proportion of variance of each node explained by the neighboring nodes in the network (see [56].) Though similar effects can be achieved with our using of bootstrapping, we estimated the predictability index of our analysis using the *mgm* package in *R* (see Additional file 1: Appendix C in Supplementary Materials for further description and results.)

Next, we tested the stability of the centrality indices with case-dropping bootstrapping [48], which is the process of repeatedly estimating a model while dropping rows of the data (i.e., we measure stability while only observing subsets of the data.) We calculated a correlation-stability (CS) coefficient, which indicates the maximum proportion of the data that can be dropped while continuing to estimate centrality values that correlate highly (*r* > .7) with the network from the full sample. Scores .25 and .5 indicate benchmarks for adequate and good network stability, respectively [48]. For each network, we created plots displaying the CIs of edges and centrality values, as well as their confidence intervals. Bootstrapped difference plots are useful for estimating which edges or centrality values can be meaningfully interpreted as distinct; we used these plots to guide our interpretations of edge and centrality values.

#### Bridge nodes

In a network with multiple scales, bridge nodes are the main nodes that connect to other node clusters, in this case––the other psychiatric and wellbeing measures. We can find these bridge nodes by calculating the bridge centrality statistics from the *networktools* package in *R* [47]. The bridge centrality statistic applies to a node’s connection to all the other nodes in the other communities to which it does not belong. Bridge strength is defined as the sum of the absolute value of all edges that exist between a given node and all nodes that are not in the same community. Bridge expected influence (one-step) is defined as the sum of the value (either positive or negative) of all edges that exist between a node and all nodes that are in a different community than the node [47, 55]. Thus, bridge expected influence accounts for how positive and negative edges can neutralize each other. For example, if *fasting* is positively linked to *desire for thinness* (*r* = .6) and negatively to *binge eating* (*r* = −.3), then the regular strength centrality for *fasting* will be 0.9 (sum of absolute values of those edges). However, the bridge expected influence for *fasting* would be 0.3 because the value of the negative edge is subtracted from the value of the positive edge. Thus, high bridge expected value would indicate that the node is strongly and positively connected to other nodes.

To find bridge nodes, we defined two community clusters in our network. The first community, adolescent psychopathology, included the depression and anxiety symptoms, and the second community, psychological wellbeing, included the gratitude, happiness, optimism, social support, and perceived control items.

## Results

### Descriptive statistics and prevalence

The mean, standard deviation, minimum, maximum, skewness, and kurtosis for both depression and anxiety symptoms are reported in Table 2. The overall mean of the total scores for PHQ and GAD were below the moderate-to-severe cutoff of 10 (PHQ = 7.96; GAD = 7.46). Some 28.56% of participants endorsed symptoms of moderate-to-severe depression. A higher 26.55% endorsed symptoms of moderate-to-severe anxiety. For depression, the symptoms with the highest mean ratings were PHQ6 *(self-blame)* and PHQ7 *(trouble concentrating),* while those for anxiety were GAD3 *(too much worry)* and GAD2 *(uncontrollable worry).* The descriptive statistics for the EPOCH, MSSS, PCS, and GQ-6 items are also reported in Table 2.

**Table 2:** Mean, standard deviation, minimum, maximum, skewness, and kurtosis of PHQ and GAD symptoms and EPOCH, MSSS, GQ-6 and PCS items

Variable	M	SD	Min	Max	Skewness	Kurtosis	Variable	M	SD	Min	Max	Skewness	Kurtosis
PHQ1: Little interest/pleasure	1.13	1.00	0	3	0.67	− 0.56	MSSS3: Family helps me	6.14	1.31	1	7	−2.10	4.52
PHQ2: Depressed mood	0.95	0.96	0	3	0.86	−0.18	MSSS4: Family provides emotional help and support	5.43	1.69	1	7	−1.15	0.48
PHQ3: Sleep problems	1.00	1.08	0	3	0.76	−0.73	MSSS5: I have a special person is a real source of comfort	5.23	1.80	1	7	−0.92	−0.17
PHQ4: Little energy	0.89	0.92	0	3	0.89	−0.02	MSSS6: Friends try to help me	4.70	1.61	1	7	−0.68	−0.14
PHQ5: Appetite problems	0.83	1.04	0	3	1.02	−0.26	MSSS7: I can count on friends	4.35	1.75	1	7	−0.44	−0.75
PHQ6: Self-blame	1.25	1.13	0	3	0.42	−1.22	MSSS8: I can talk to family about problems	4.77	1.92	1	7	−0.61	−0.79
PHQ7: Trouble concentrating	1.20	1.08	0	3	0.50	−1.00	MSSS9: I have friends to share joys/sorrows	4.88	1.71	1	7	−0.83	−0.13
PHQ8: Psychomotor problems	0.71	0.99	0	3	1.24	0.32	MSSS10: I have a special person who cares about my feelings	5.41	1.77	1	7	−1.12	0.33
GAD1: Nervousness	1.10	1.01	0	3	0.63	−0.66	MSSS11: Family willing to help me make decisions	5.76	1.52	1	7	−1.56	1.97
GAD2: Uncontrollable worry	1.26	1.20	0	3	0.42	−1.12	MSSS12: I can talk about my problems with friends	4.30	1.77	1	7	−0.40	−0.79
GAD3: Too much worry	1.31	1.26	0	3	0.38	−1.18	GQ1: I have a lot to be thankful for	6.39	1.06	1	7	−2.79	9.24
GAD4: Trouble relaxing	0.89	0.73	0	3	0.92	−0.22	GQ2: I do not see a lot to be thankful for	5.75	1.55	1	7	−1.40	1.13
GAD5: Restlessness	0.60	0.42	0	3	1.47	1.28	GQ3: I am grateful to different people	6.04	1.26	1	7	−1.95	4.05
GAD6: Irritability	1.08	0.98	0	3	0.67	−0.82	GQ4: I don’t feel grateful often	5.08	1.78	1	7	−0.71	−0.74
GAD7: Feeling afraid	1.16	1.07	0	3	0.57	−0.92	GQ5: I appreciate things in the past	5.78	1.58	1	7	−1.65	1.99
O1: Optimistic about future	3.68	1.19	1	5	−0.44	−0.97	GQ6: I can write down a long list of things to be grateful for	5.98	1.38	1	7	−1.82	3.09
O2: Expect the best	3.14	1.22	1	5	0.15	−1.11	PCS1: I can get good grades if I try	2.84	0.45	0	3	−3.37	13.21
O3: Good things will happen to me	3.63	1.19	1	5	−0.40	−0.95	PCS2: I can do well on tests if I study	2.80	0.50	0	3	−3.10	11.13
O4: Things will work out no matter what	3.69	1.24	1	5	−0.49	−0.97	PCS3: I can get good marks on homework if I work at it	2.76	0.56	0	3	−2.85	8.95
H1: I feel happy	3.28	1.16	1	5	0.13	−1.15	PCS4: I cannot succeed at school no matter how I try	2.70	0.71	0	3	−2.62	6.18
H2: I have a lot of fun	2.87	1.15	1	5	0.45	−0.74	PCS5: I cannot get good grades no matter how I try	2.69	0.69	0	3	−2.51	5.70
H3: I love life	3.66	1.27	1	5	−0.45	−1.06	PCS6: I cannot do well at tests no matter how hard I try	2.69	0.66	0	3	−2.35	5.01
H4: I am a joyful person	3.48	1.20	1	5	−0.17	−1.15	PCS7: I cannot get good marks for my homework even if I work hard at it	2.68	0.68	0	3	−2.45	5.61
MSSS1: Special person around for me	5.24	1.63	1	7	−0.92	0.17	PCS8: I can succeed in school if I try	2.76	0.66	0	3	−3.16	9.56
MSSS2: Special person to share joy/sorrows	5.23	1.73	1	7	−0.98	0.04							

*PHQ-8* Patient Health Questionnaire, *GAD-7* Generalized Anxiety Disorder Screener, *EPOCH* EPOCH Measure of Adolescent of Well-Being, *MSSS* Multidimensional Scale of Perceived Social Support, *PCS* Perceived Control Scale, *GQ* Gratitude Questionnaire

### Network analysis: adolescent psychopathology and psychological wellbeing

#### Node selection

Conducting the *goldbricker* procedure, we found nine pairs of symptoms with less than 20% significantly different correlations (see Table 3). We first removed the symptoms that were redundant with at least two other symptoms. In other words, we removed MSSS6 (*friends try to help me*), MSSS5 (*I have a special person who is a real source of comfort to me*), and PCS5 (*I cannot get good grades no matter how hard I try*), which allows us to keep MSSS7, MSSS2, MSSS9, MSSS10, PCS4, and PCS7 in the network unaltered. The remaining overlapping symptom pairs were GAD2–3 (*uncontrollable worry––too much worry*), PCS1–2 (*I can get good grades if I try––I can do well on tests if I study*), and GQ1–6 (*I have a lot to be thankful for––I can write down a long list of things to be grateful for*), and we decided to keep GAD2, PCS1, and GQ1 in our network.

**Table 3:** Variable Pairs with < 20% Different Correlations

Variable 1	Variable 2	Different Correlations (%)
MSSS6: My friends really try to help me	MSSS7: I can count on my friends when things go wrong	8.51
GAD2: Uncontrollable worry	GAD3: Too much worry	8.51
MSSS2: There is a special person with whom I can share my joys and sorrows	MSSS5: I have a special person who is a real source of comfort to me	14.89
PCS1: I can get good grades if I really try.	PCS2: I can do well on tests at school if I study hard.	14.89
MSSS6: My friends really try to help me	MSSS9: I have friends with whom I can share my joys and sorrows	17.02
MSSS5: I have a special person who is a real source of comfort to me	MSSS10: There is a special person in my life who cares about my feelings	17.02
PCS4: I cannot succeed at school no matter how hard I try.	PCS5: I cannot get good grades no matter how hard I try.	19.15
PCS5: I cannot get good grades no matter how hard I try.	PCS7: I cannot get good marks for my homework, even if I work hard at it.	19.15
GQ1: I have a lot to be thankful for in my life	GQ6: I can write down a long list of things to be grateful for	19.15

*PHQ-8* Patient Health Questionnaire, *GAD-7* Generalized Anxiety Disorder Screener, *MSSS* Multidimensional Scale of Perceived Social Support, *PCS* Perceived Control Scale, *GQ* Gratitude Questionnaire

#### Network estimation

**Fig. 1:**
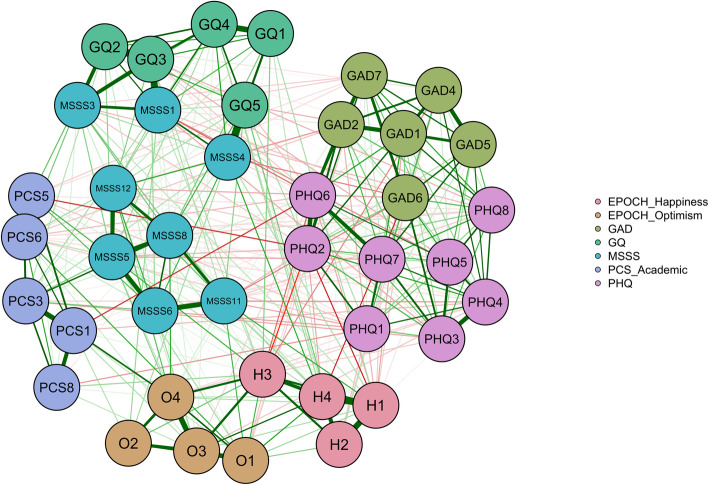
Regularized partial correlation network for all measures of adolescent psychopathology and wellbeing

The network for all seven measures was connected, see Fig. 1. The psychometric associations, or edge-values, between 43 nodes were calculated (see Additional file 1: Appendix D in Supplementary Materials.) The mean weight of edges was 0.0177, and 378 of the 903 edges were non-zero edges. The edges revealed positive associations within both wellness measures and mental disorder measures, but negative associations between the two groups of constructs. All within-measure items were consistently clustered together. In other words, when visualized, the networks revealed that each measure formed its own cluster. Notably, the symptoms of PHQ-8 and GAD-7 had strongest inter-measure connections, even appearing to overlap and form one large cluster in the plotted network. Among the psychological wellbeing measures, the EPOCH subscales of optimism and happiness were the most highly connected, and they also had strong edges with other wellbeing scales of perceived academic control, gratitude, and social support. We found that the network with all the measures had a network density of 46%, which represents the percentage of nonzero edges estimated over the total number of possible edges.

We also investigated the interconnectivity between the two constructs. The strongest negative edges across psychological wellbeing measures and symptoms of depression were PHQ2––H3 *(depressed mood—I love life)*, PHQ2––H1 *(depressed mood—I feel happy)*, PHQ6––PCS1 *(self-blame—I can get good grades if I try),* PHQ6––MSSS8 *(self-blame—I can talk to family about things)*, and PHQ6––H3 *(self-blame—I love life.)* Meanwhile, those with symptoms of anxiety were GAD6––H4 *(irritability—I am a joyful person)*, GAD6––MSSS12 *(irritability—I can talk to friends about problems)*, and GAD6––GQ4 *(irritability—I don’t feel grateful)*.

#### Centrality

**Fig. 2:**
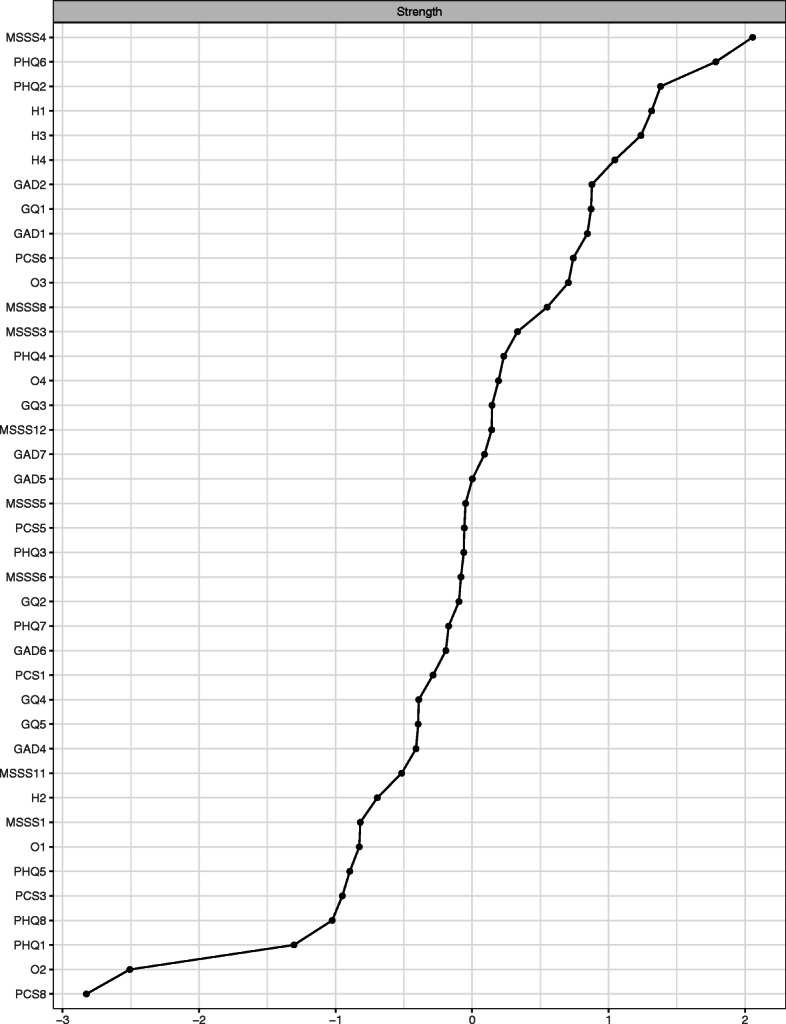
Symptom centrality (strength) in a network of all measures of adolescent psychopathology and wellbeing

The most highly central symptoms according to *strength* (see Fig. 2) were *MSSS4 (family provides emotional help and support),* PHQ6 *(self-blame),* and PHQ2 *(depressed mood).* The following symptoms were the next highest centrality statistics: H1 *(I feel happy),* H3 *(I love life),* H4 *(I am a joyful person),* GAD2 *(uncontrollable worry)*, GQ1 *(I have a lot to be thankful for),* GAD1 *(nervousness),* and PCS6 (*I cannot do well at tests no matter how hard I try*.) Thus, three of the four happiness measures were in the top ten most central symptoms. Furthermore, we determined these symptom items with significantly higher *strength* values by performing a difference test between their centrality statistics, using nonparametric bootstrapping with *bootnet* [48]. On the other end, the least central symptoms according to *strength* were PCS8 *(I can succeed in school if I try)*, O2 *(expect the best)*, and PHQ1 *(little interest/pleasure.)*

#### Bridge nodes

**Fig. 3:**
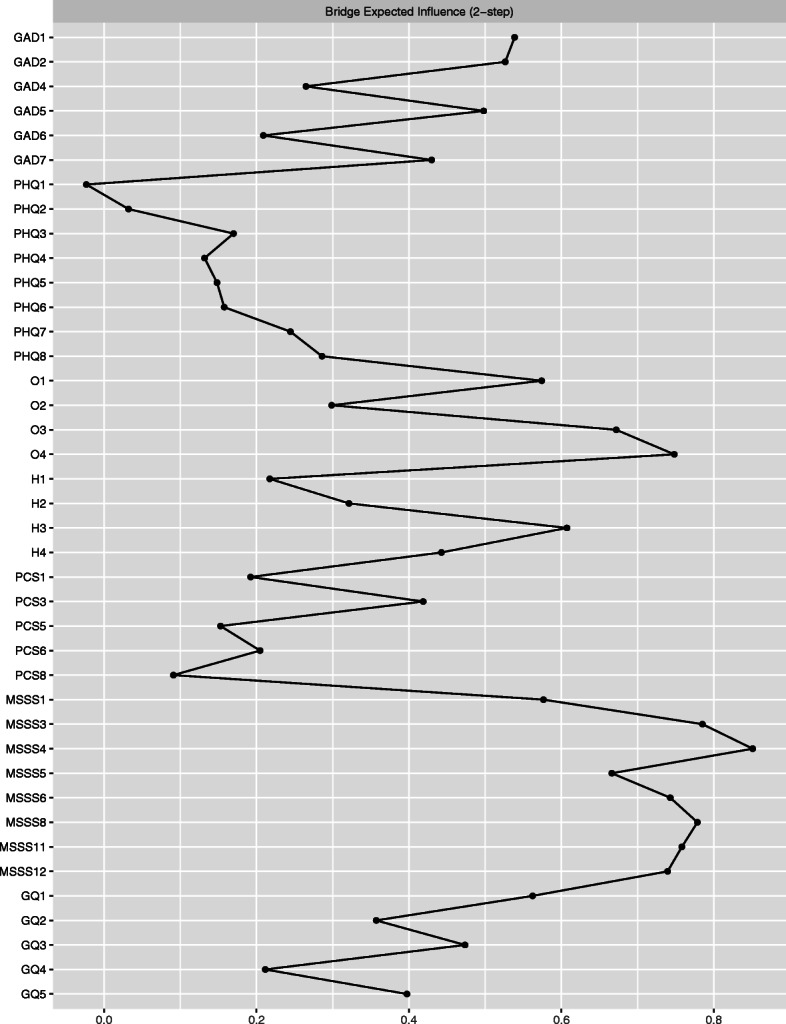
Bridge items expected influence in a network with two communities for adolescent psychopathology and psychological wellbeing

To dig deeper into the community clusters form and to investigate the ties between wellbeing and symptoms of depression and anxiety, we defined two communities, one for psychopathology and the other for psychological wellbeing. We were then able to investigate the bridge symptoms between these two clusters. Calculating the bridge *expected influence* (the bridge statistic that neutralizes positive and negative edges) and bridge *strength*, we identified the nodes that were most strongly and positively connected to nodes from all other measures. As can be seen in Fig. 3, the main bridge nodes were all from the Multidimensional Scale of Perceived Social Support: MSSS3 *(family helps me)*, MSSS8 *(I can talk to family about problems)*, MSSS11 *(family willing to help me make decisions)*, O4 *(things will work out no matter what),* and MSSS12 *(I can talk about my problems with friends.*) The most negative bridge node was PHQ1 *(little interest/pleasure), followed by* PHQ2 *(depressed mood)* and PCS8 *(I can succeed in school if I try.)* Additionally, we ran bootstraps to find a good bridge strength stability with the maximum drop proportion of 0.854 to retain a correlation of 0.7 in at least 95% of the samples.

#### Network accuracy and stability

The bootstrapped 95% CIs around the edge-weights were not large, meaning that the edges did not significantly vary across the bootstraps. The small CIs indicate that the edge-weights are reliable and accurate (for CI plots, see Additional file 1: Appendix B in Supplementary Materials.) The network also had high stability of centrality. We ran a “case dropping” bootstrap, such that we incrementally reduced the sample to determine how small we can reduce it while still maintaining a stable network. Using 15,000 bots (i.e., reduce sample, estimate network, and compute centralities fifteen thousand times), we found that 87% of the sample could be dropped before the correlation with centrality values of the full dataset dropped below *r* = .7 in at least 95% of the samples.

## Discussion

We conducted a network analysis to analyze the structure of psychological wellbeing indicators and symptoms of depression and anxiety in a greatly understudied population in Sub Saharan Africa––a large community sample of Kenyan adolescents. Our results, which were robust to statistical and accuracy tests, revealed how indicators of psychological wellbeing (like happiness and gratitude) and psychopathology measures of depression and anxiety clustered in a network. We also identified the central features of adolescent psychopathology and wellbeing, as well as the interconnectedness of the various items within these domains.

This study is, to the best of our knowledge, the first attempt of its kind to combine wellbeing elements and psychopathology in a network approach with SSA youths. The recent network research of child and adolescent psychopathology has been conducted almost exclusively with Western adolescent populations [52, 57–60] with only a few studies in the Global South [61–63]. More research focused on SSA youths is necessary since there are scientific benefits of cross-cultural research and culture affects psychopathology and psychological wellbeing. Additionally, there is the potential utility of such research in SSA where prevalence rates for mental disorders are high [2, 21], stigma inhibits help-seeking [23], mental healthcare infrastructure is poor [2], and a large percentage of the population is youthful (the mean age in Kenya is 19.4 years [64].) Thus, our results expand our understanding of psychological wellbeing in relation to adolescent psychopathology in a hitherto understudied and at-risk population.

In our psychological well-being, depression, and anxiety network, two distinct clusters emerged. The constructs of psychological wellbeing (gratitude, happiness, optimism, social support, and perceived control) clustered together, while the constructs of adolescent psychopathology (depression and anxiety) formed a separate community in the network. Thus, our findings offer support for the dual factor model of psychopathology and psychological wellbeing, in which the two concepts are distinct but related constructs [8, 19]. We found that the elements of psychopathology and those of psychological wellbeing formed two distinct clusters that were strongly and negatively associated with each other. Within the positive psychological wellbeing cluster, we found each of the constructs of psychological wellbeing to form distinct but closely connected clusters. This suggests that happiness, optimism, gratitude, and perceived control are separate but closely related constructs of psychological wellbeing. Happiness and optimism were highly connected, perhaps due to their overlapping notions or perhaps since they were subscales of the same index. Similarly, within the psychopathology cluster, anxiety and depression did not overlap but formed separate yet closely related clusters.

The wellbeing items––*family provides emotional help and support, I feel happy, I love life, I am a joyful person,* and *I have a lot to be thankful for––*as well as the psychopathology items–*–self-blame, depressed mood, uncontrollable worry, nervousness,* and *I cannot do well at tests no matter how hard I try––*were the most central nodes in our network of psychological wellbeing and adolescent psychopathological, according to *strength*. This suggests that these items, which represent a diversity of items from different measures, may be especially important because they are strongly connected to other symptoms. Specifically, the two most central nodes in the network were *family provides emotional help and support* and *self-blame*. Emotional help and support from family and loved ones during this development period appear to be key to tackle negative psychopathological symptoms. As shown in Fig. 2, three of the four happiness items were in the top seven most central nodes, suggesting the potential utility of using happiness traits to uncover and prevent mental health symptoms.

Additionally, we defined two communities for the psychological wellbeing and adolescent psychopathology measures. The social support items––*family helps me, I can talk to family about problems,* and *family willing to help me make decisions––*were the most important bridge nodes that connected the two community clusters*.* In addition, *nervousness* was the most important psychopathology bridge node between the two clusters.

It is particularly striking that four of the top five strongest bridge nodes were social support items. The association between social support and mental health problems, as well as the interpretation of social support as an important protective factor against depression and anxiety, has been documented extensively elsewhere [65, 66]. In the context of Kenyan youths, it is critical to view social support in light of the nature of the Kenyan educational system. Indeed, some observers have pointed out that rather than social support, many Kenyan youths experience increased psychosocial pressure from their families, friends, and loved ones to do well in the end-of-secondary school examinations [67]. As these examinations are important in determining future prospects, the external pressure to succeed from friends and families has been linked with increased depressive and anxiety symptoms amongst Kenyan youths [32]. Future studies should replicate these findings, as the association between academics, social support, and adolescent psychopathology could potentially have important public policy implications.

It is worth highlighting that *little interest/pleasure* emerged as the most negative bridge node. While further investigations are required to explore the means through which this symptom affects the relationship between positive wellbeing and psychopathology, one can imagine that having *little interest/pleasure* in everyday things may lead adolescents to live withdrawn lives that are absent of social support needed to improve positive wellbeing.

While these findings are insufficient to draw claims about interventions, they may suggest why positive psychological interventions that target psychological wellbeing elements rather than psychopathology—such as the *Shamiri* (“thrive”) intervention [30]—have been successful in treating depression and anxiety symptoms with Kenyan youths. For example, research on trait gratitude suggests that having a lot of things to be grateful for is associated with exhibiting positive states and outcomes that may buffer against depression and anxiety [68]. Perhaps interventions that make salient elements of psychological wellbeing like gratitude may be effective in reducing youth depression and anxiety symptoms [31, 36, 37] because they target central elements in the network of wellbeing and psychopathology (e.g., *I have a lot to be grateful for*). Further studies are required to investigate this proposition, which may be particularly promising for SSA regions where social stigma around psychopathology might inhibit help-seeking.

An important strength of our study is the large sample size; however, the network connectivity that we report might not be generalized across different samples in SSA. In addition, our use of LASSO regularization to reduce false positives—which is the current “norm of practice” in many similar studies (see [52, 53], for example)—limits our ability to interpret our findings as independent of the sample. While we use this regularization technique to offer opportunities for comparison of our findings with those in the literature, it may be valuable for future research that uses network analysis to adopt regularization techniques that lend themselves to generalizability. That said, this weakness should be considered within the broader context of there being very few attempts to describe adolescent psychopathology and psychological wellbeing with a sample from SSA.

Another limitation is that we use measures that have minimal previous use with Kenyan youths. Though the psychometric properties of these studies have been studied and validated elsewhere, it is important that future studies use more culturally apt and psychometrically robust measures to replicate our findings. Finally, another limitation is that our study does not address the complicated clustering of our data or the role of sociodemographic variables in the network between adolescent psychopathology and wellbeing. We investigated the network differences and similarities between urban and rural subgroups (see Additional file 1: Appendix A in the Supplementary Materials.) Future studies are required to investigate this.

## Conclusions

Our study used network analysis to investigate the relationship between youth psychopathology and psychological wellbeing in a large community sample of SSA youths. Our results suggested that adolescent psychopathology and psychological wellbeing are two distinct but related constructs. We identified important central and bridge nodes in the networks of psychopathology and psychological wellbeing. These results not only expand our understanding of the relationship between wellbeing and psychopathology in an understudied population, but they are also suggestive of future research directions that can espouse how the relationship between these two constructs can inform preventive and therapeutic efforts in low-income regions such as SSA.

## Supplementary Information

**Additional file 1.**

## Data Availability

All data used for the present study is stored in the Open Science Framework repository and is publicly available (DOI: 10.17605/OSF.IO/8M5D9).

## Abbreviations

PHQ-8: Patient Health Questionnaire-8
GAD-7: Generalized Anxiety Disorder Screener-7
MSSS: Multidimensional Scale of Perceived Social Support
PCS: Perceived Control Scale
GQ-6: Gratitude Questionnaire – 6
EPOCH: Engagement, Perseverance, Optimism, Connectedness, and Happiness Scale
MUERC: Maseno University Ethics Review Committee
