# Supplementary material for: Adolescent psychopathology and psychological wellbeing: a network analysis approach
Source: BMC Psychiatry. 2021 Jul 3;21:333. doi: 10.1186/s12888-021-03331-x (PMC8254923; doi:10.1186/s12888-021-03331-x)
Supplement: Supplementary file 1 — Additional file 1. [file 12888_2021_3331_MOESM1_ESM.docx]

**APPENDIX A: Network Differences and Similarities Between Urban and Rural Subgroups**

The 2,192 Kenyan adolescents recruited as part of a large-scale clinical trial (called *Shamiri* [30,31]) were students at four secondary schools in Nairobi and Kiambu Counties. Two of which were urban schools and two were rural. We investigated these two local clusters and found that the network for rural schools and the one for the urban schools had many similarities, as well as some differences. We have included an overview of their structural analysis here. That said, a careful investigation of how the different community clusters affect the network structure, as well as the role of factors such as community risk, SES, and religious externalities would beyond the scope of our present study.


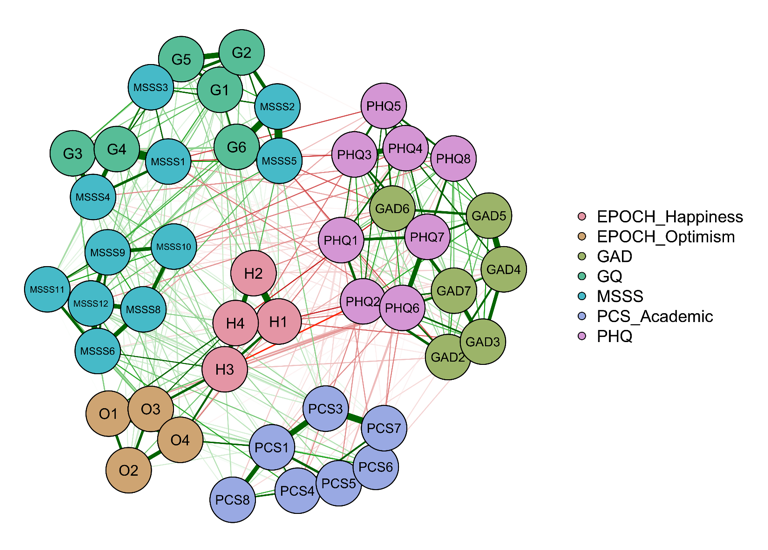


*Figure S1: Urban school network*


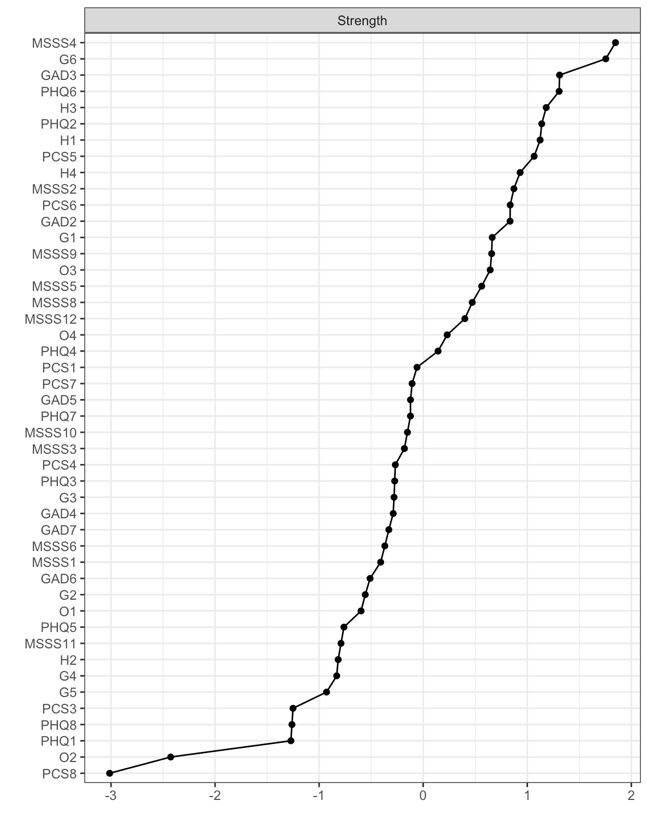


*Figure S2: Centrality (strength) for urban school network*

The *urban network* consisted of more participants (*N* = 1,716) of which 26.11% (*N* = 448) were above the PHQ threshold of 10 and 24.77% (*N* = 425) were above the GAD threshold of also 10. We removed the redundant nodes GAD1 (*nervousness*), PCS2 (*I can do well on tests if I study*), and MSSS7 (*I can count on friends*) and then estimated the network (see Figure S1.) We found the most central symptom according to Strength (see Figure S2) to be MSSS4 (*Family provides emotional help and support.)*


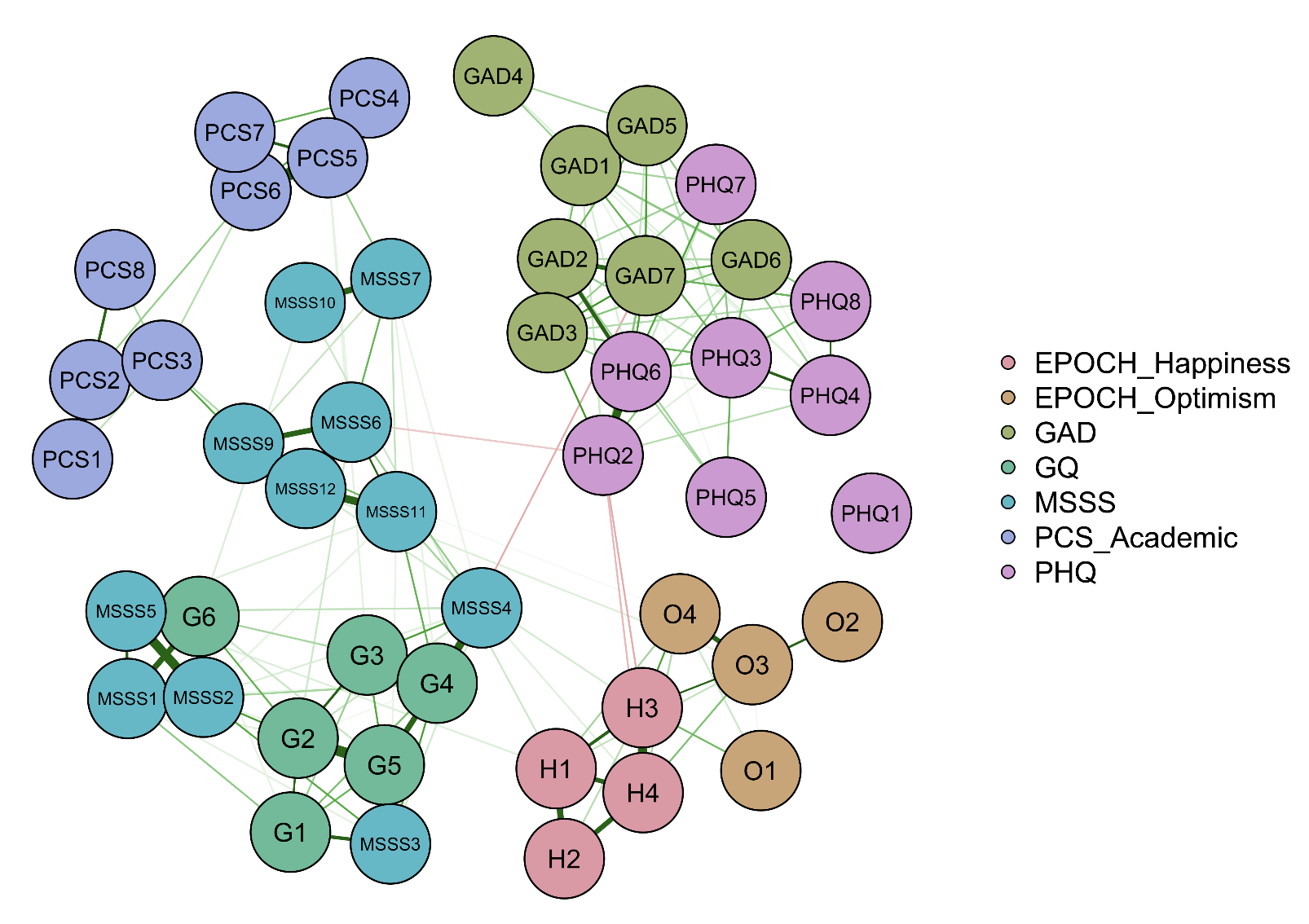


*Figure S3: Rural school network*


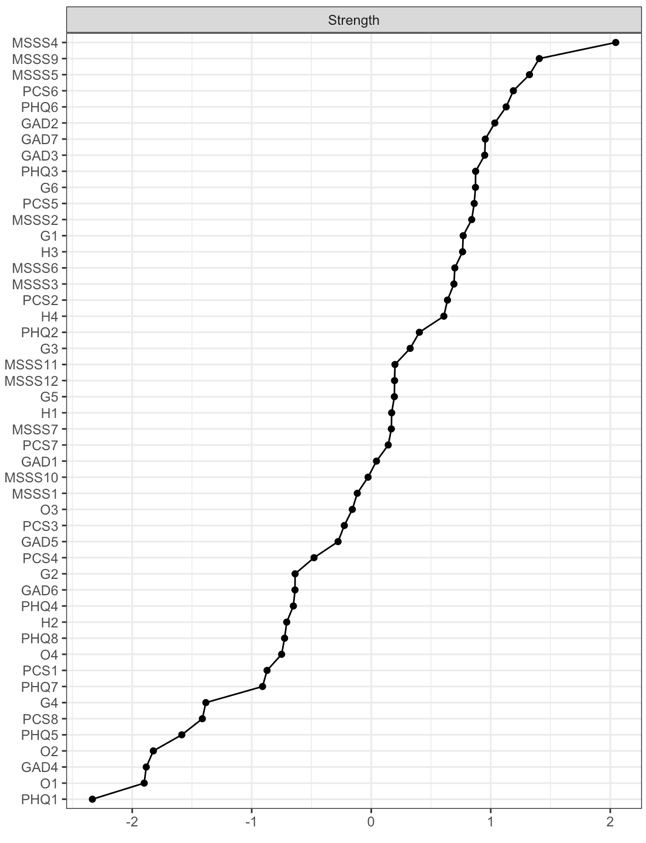


*Figure S4: Centrality (strength) for rural school network*

The *rural network* consisted of smaller proportion of participants (*N* = 476) of which more reached the depression and anxieties cutoffs of 10. Specifically, 37.40% (*N* = 178) were above the PHQ threshold and 32.98% (*N* = 157) for GAD. We removed only one redundant node: MSSS8 (*I can talk to family about problems*) and then estimated the network (see Figure S3.) As with the *urban network*, we found the most central symptom according to Strength (see Figure S4) to be MSSS4 (*Family provides emotional help and support.)*

**APPENDIX B: Confidence Intervals of Edge-Weights**

As described in the paper, we implement a bootstrapping strategy using the *bootnet* package in R to test the centrality stability via a case-dropping estimation (i.e., we measured stability while only observing subsets of the data.) We calculated a correlation-stability (CS) coefficient, which indicates the maximum proportion of the data that can be dropped while continuing to estimate centrality values that correlate highly (r > .7) with the network from the full sample. Scores .25 and .5 indicate benchmarks for adequate and good network stability, respectively [47]. These centrality values are considered unstable when the correlations drop significantly across different subsamples. For each network, we created plots displaying the confidence intervals (CIs) of edges (see Figure S5) and centrality values according to the *strength* statistic, as well as their confidence intervals.


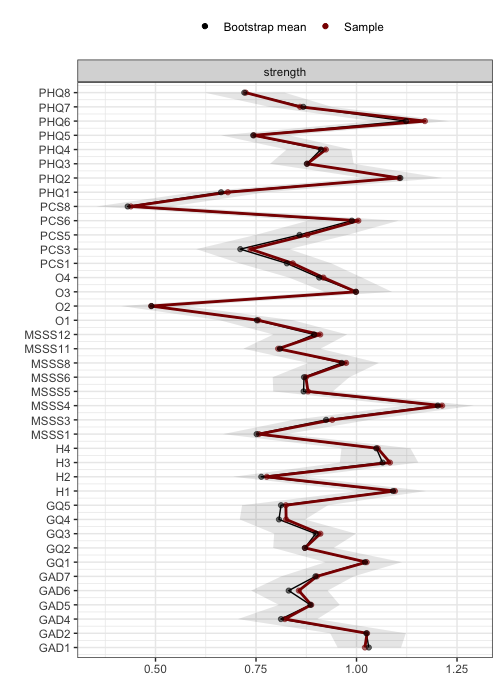


*Figure S5: Bootstrapped values for the edge weights.*

We plotted the CIs using the *bootnet* package and, specifically, the *plot=”area”* function keyword to produce a graph with a shaded area indicating the confidence region of the edge-weights for the estimated network. Specifically, *red line* on the graph represents the sample values, the *black line* indicates the bootstrapped mean, and the *grey shaded area* depicts the bootstrapped confidence intervals. These CIs show that the network has good stability.

**APPENDIX C: Predictability Indices**

By analyzing the structure of the network, we found that the most highly central symptoms according to *strength* (see Figure 2) were *MSSS4 (family provides emotional help and support),* PHQ6 *(self-blame),* and PHQ2 *(depressed mood).* When interpreting centrality measures such as these, we must also consider the degree to which each symptom can be determined by the others in the network. Thus, we calculated the node predictability by estimating the network using the GGM model with the *mgm* package in R (see Figure S6) and calculating the predictability estimation (Haslbeck & Waldorp, 2018).


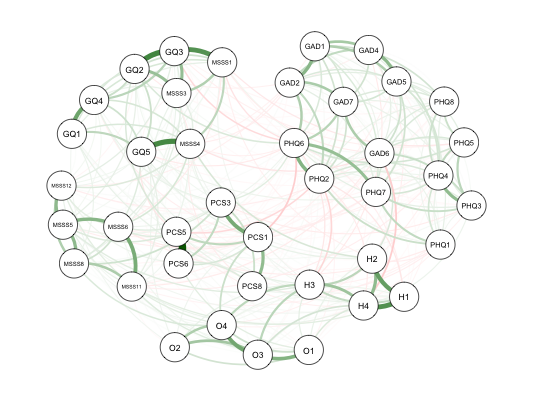


Figure S6: Estimated network using *mgm* package.

Node predictability goes beyond the relative importance of nodes as it measures the proportion of variance of each node that is explained by neighboring nodes in the network (between 0 and 1) and, thus, represents interconnectedness of each given node. At the extremes, nodes with a predictability index of 0 cannot be explained by the network model, while those with a value of 1 can be perfectly predicted by it. The estimated predictability indices for the nodes in the network were determined (see Table S1.)

|  | **Predictability Index** |
| --- | --- |
| **GAD1** | 0.397 |
| **GAD2** | 0.408 |
| **GAD4** | 0.313 |
| **GAD5** | 0.326 |
| **GAD6** | 0.267 |
| **GAD7** | 0.337 |
| **PHQ1** | 0.188 |
| **PHQ2** | 0.401 |
| **PHQ3** | 0.265 |
| **PHQ4** | 0.298 |
| **PHQ5** | 0.233 |
| **PHQ6** | 0.432 |
| **PHQ7** | 0.298 |
| **PHQ8** | 0.218 |
| **O1** | 0.259 |
| **O2** | 0.166 |
| **O3** | 0.368 |
| **O4** | 0.323 |
| **H1** | 0.473 |
| **H2** | 0.362 |
| **H3** | 0.394 |
| **H4** | 0.463 |
| **PCS1** | 0.269 |
| **PCS3** | 0.221 |
| **PCS5** | 0.422 |
| **PCS6** | 0.427 |
| **PCS8** | 0.123 |
| **MSSS1** | 0.296 |
| **MSSS3** | 0.457 |
| **MSSS4** | 0.554 |
| **MSSS5** | 0.354 |
| **MSSS6** | 0.343 |
| **MSSS8** | 0.437 |
| **MSSS11** | 0.348 |
| **MSSS12** | 0.342 |
| **G1** | 0.354 |
| **G2** | 0.312 |
| **G3** | 0.300 |
| **G4** | 0.266 |
| **G5** | 0.270 |

Table S1: Node predictability indices calculated using *mgm* package.

This network results in similar associations, and the nodes had significant variance explained by their neighboring nodes. The predictability index indicates that, on average across all nodes, 33.21% of a node’s variance is explained by its neighboring nodes. The node with the highest predictability score was MSSS4 *(family provides emotional help and support)* with 55.4% of variance explained by surrounding nodes. *(family provides emotional help and support),* H1 *(I feel happy)*, H4 *(I am a joyful person)*, and MSSS3 *(family helps me)* had over 45% of variance explained.

**APPENDIX D: Network Edge-Values**

In this paper, our network, which was estimated using a regularized Gaussian graphical model, consists of 43 symptoms (or nodes) and the psychometric associations between them. We decided to explicitly include these symptom associations (or edge-values) as tables below. The mean weight of edges was 0.0177, and 378 of the 903 edges were non-zero edges.

|  | **GAD1** | **GAD2** | **GAD4** | **GAD5** | **GAD6** | **GAD7** |
| --- | --- | --- | --- | --- | --- | --- |
| **GAD1** | - | 0.2048 | 0.1559 | 0.1389 | 0.0349 | 0.0717 |
| **GAD2** | 0.2048 | - | 0.1064 | 0.0645 | 0.0274 | 0.1456 |
| **GAD4** | 0.1559 | 0.1064 | - | 0.1965 | 0.0382 | 0.0812 |
| **GAD5** | 0.1389 | 0.0645 | 0.1965 | - | 0.0807 | 0.0755 |
| **GAD6** | 0.0349 | 0.0274 | 0.0382 | 0.0807 | - | 0.1419 |
| **GAD7** | 0.0717 | 0.1456 | 0.0812 | 0.0755 | 0.1419 | - |
| **PHQ1** | 0.0258 | 0.0074 | 0.0453 | 0.0145 | 0.0277 | 0.0085 |
| **PHQ2** | 0.0803 | 0.1060 | 0 | 0.0200 | 0.0364 | 0.0826 |
| **PHQ3** | 0.0234 | 0.0364 | 0 | 0.0232 | 0.0996 | 0 |
| **PHQ4** | 0.0269 | 0 | 0.0713 | 0.0684 | 0.0223 | 0 |
| **PHQ5** | 0.0060 | 0.0095 | 0.0127 | 0.0485 | 0.0640 | 0.0363 |
| **PHQ6** | 0.0638 | 0.1344 | 0.0112 | 0 | 0.0194 | 0.1194 |
| **PHQ7** | 0.0629 | 0.0498 | 0.0120 | 0.0809 | 0 | 0.0153 |
| **PHQ8** | 0.0760 | 0.0452 | 0.0144 | 0.0456 | 0.0484 | 0.0597 |
| **O1** | 0 | 0 | -0.0016 | 0 | 0 | -0.0132 |
| **O2** | 0 | 0 | 0 | 0 | 0 | 0 |
| **O3** | 0 | 0 | 0 | 0 | 0 | 0 |
| **O4** | 0 | 0 | 0 | 0 | 0 | 0 |
| **H1** | -0.0253 | -0.0187 | 0 | -0.0062 | -0.0089 | 0 |
| **H2** | 0 | 0 | -0.0107 | 0 | 0.0020 | 0 |
| **H3** | -0.0014 | -0.0055 | -0.0331 | 0 | 0 | 0 |
| **H4** | 0 | 0 | 0 | 0 | -0.0549 | -0.0050 |
| **PCS1** | 0 | 0 | 0 | 0 | -0.0047 | 0 |
| **PCS3** | 0 | 0 | 0 | 0 | 0 | 0 |
| **PCS4** | 0 | 0 | -0.0071 | 0 | 0 | 0 |
| **PCS6** | 0 | 0 | -0.0123 | 0 | 0 | -0.0130 |
| **PCS7** | 0 | 0 | 0 | 0 | 0 | 0 |
| **PCS8** | 0 | 0 | 0 | 0 | 0 | 0 |
| **MSSS1** | 0 | 0 | 0 | 0 | 0 | 0 |
| **MSSS2** | 0 | 0 | 0 | 0 | 0 | 0 |
| **MSSS3** | 0 | 0 | -0.0040 | 0 | 0 | 0 |
| **MSSS4** | -0.0066 | 0 | -0.0030 | 0 | 0 | 0 |
| **MSSS7** | 0 | -0.0231 | 0 | 0 | -0.0023 | 0 |
| **MSSS8** | -0.0016 | -0.0046 | 0 | 0 | -0.0160 | -0.0134 |
| **MSSS9** | 0 | 0 | 0 | 0 | 0 | 0 |
| **MSSS10** | 0 | 0 | 0 | 0 | 0 | 0 |
| **MSSS11** | 0 | 0 | 0 | -0.0005 | 0 | -0.0125 |
| **MSSS12** | 0 | -0.0196 | 0 | 0 | -0.0321 | 0 |
| **G1** | 0 | 0 | 0 | 0 | 0 | 0 |
| **G2** | 0 | 0 | 0 | 0 | 0 | 0 |
| **G3** | 0 | 0 | 0 | 0 | -0.0052 | 0 |
| **G4** | -0.0024 | -0.0022 | 0 | -0.0191 | -0.0346 | 0 |
| **G5** | 0 | -0.0110 | 0 | 0 | -0.0233 | 0 |

*Table S1. Edge-weights for GAD.*

|  | **PHQ1** | **PHQ2** | **PHQ3** | **PHQ4** | **PHQ5** | **PHQ6** |
| --- | --- | --- | --- | --- | --- | --- |
| **GAD1** | 0.0258 | 0.0803 | 0.0234 | 0.0269 | 0.0060 | 0.0638 |
| **GAD2** | 0.0074 | 0.1060 | 0.0364 | 0 | 0.0095 | 0.1344 |
| **GAD4** | 0.0453 | 0 | 0 | 0.0713 | 0.0127 | 0.0112 |
| **GAD5** | 0.0145 | 0.0200 | 0.0232 | 0.0684 | 0.0485 | 0 |
| **GAD6** | 0.0277 | 0.0364 | 0.0996 | 0.0223 | 0.0640 | 0.0194 |
| **GAD7** | 0.0085 | 0.0826 | 0 | 0 | 0.0363 | 0.1194 |
| **PHQ1** | - | 0.0933 | 0.0397 | 0.0724 | 0.0355 | 0.0220 |
| **PHQ2** | 0.0933 | - | 0.0506 | 0.0623 | 0.0457 | 0.2126 |
| **PHQ3** | 0.0397 | 0.0506 | - | 0.2080 | 0.1226 | 0.0478 |
| **PHQ4** | 0.0724 | 0.0623 | 0.2080 | - | 0.1014 | 0.0150 |
| **PHQ5** | 0.0355 | 0.0457 | 0.1226 | 0.1014 | - | 0.0677 |
| **PHQ6** | 0.0220 | 0.2126 | 0.0478 | 0.0150 | 0.0677 | - |
| **PHQ7** | 0.1067 | 0.0183 | 0.1021 | 0.0698 | 0.0236 | 0.1774 |
| **PHQ8** | 0.0295 | 0.0321 | 0.0486 | 0.0826 | 0.0886 | 0 |
| **O1** | 0 | -0.0085 | 0 | 0 | 0 | -0.0153 |
| **O2** | 0 | 0 | 0 | 0 | 0 | 0 |
| **O3** | 0 | 0 | 0 | -0.0149 | 0 | -0.0105 |
| **O4** | -0.0073 | 0 | 0 | -0.0108 | 0 | 0 |
| **H1** | -0.0038 | -0.0590 | -0.0006 | -0.0031 | 0 | -0.0225 |
| **H2** | -0.0143 | 0 | 0 | 0 | 0 | 0 |
| **H3** | 0 | -0.0695 | -0.0140 | -0.0123 | 0 | -0.0398 |
| **H4** | 0 | -0.0240 | 0 | 0 | 0 | 0 |
| **PCS1** | 0 | 0 | 0 | -0.0193 | 0 | -0.0553 |
| **PCS3** | 0 | 0 | -0.0207 | -0.0046 | 0 | 0 |
| **PCS4** | 0 | -0.0445 | 0 | 0 | 0 | 0 |
| **PCS6** | -0.0220 | -0.0128 | 0 | 0 | 0 | -0.0168 |
| **PCS7** | 0 | 0 | 0 | -0.0239 | 0 | 0 |
| **PCS8** | -0.0247 | 0 | 0 | 0 | 0 | 0 |
| **MSSS1** | 0 | 0 | 0 | 0 | 0 | 0 |
| **MSSS2** | 0 | 0 | 0 | 0 | 0 | 0 |
| **MSSS3** | 0 | 0 | 0 | 0 | 0 | 0 |
| **MSSS4** | 0 | 0 | -0.0077 | 0 | -0.0219 | -0.0076 |
| **MSSS7** | -0.0040 | 0 | 0 | 0 | 0 | -0.0087 |
| **MSSS8** | 0 | 0 | -0.0231 | 0 | -0.0310 | -0.0390 |
| **MSSS9** | 0 | 0 | 0 | 0 | 0 | 0 |
| **MSSS10** | 0 | 0 | 0 | 0 | 0 | 0 |
| **MSSS11** | 0 | -0.0149 | -0.0019 | 0 | 0 | 0 |
| **MSSS12** | 0 | -0.0049 | 0 | 0 | 0 | -0.0136 |
| **G1** | 0 | -0.0239 | 0 | 0 | 0 | 0 |
| **G2** | -0.0260 | 0 | 0 | 0 | -0.0094 | 0 |
| **G3** | -0.0036 | 0 | 0 | -0.0064 | 0 | 0 |
| **G4** | -0.0235 | 0 | 0 | -0.0276 | -0.0005 | 0 |
| **G5** | -0.0072 | -0.0073 | 0 | 0 | -0.0137 | 0 |

*Table S2. Edge-weights for PHQ.*

|  | **O1** | **O2** | **O3** | **O4** | **H1** | **H2** | **H3** | **H4** |
| --- | --- | --- | --- | --- | --- | --- | --- | --- |
| **GAD1** | 0 | 0 | 0 | 0 | -0.0253 | 0 | -0.0014 | 0 |
| **GAD2** | 0 | 0 | 0 | 0 | -0.0187 | 0 | -0.0055 | 0 |
| **GAD4** | -0.0016 | 0 | 0 | 0 | 0 | -0.0107 | -0.0331 | 0 |
| **GAD5** | 0 | 0 | 0 | 0 | -0.0062 | 0 | 0 | 0 |
| **GAD6** | 0 | 0 | 0 | 0 | -0.0089 | 0.0020 | 0 | -0.0549 |
| **GAD7** | -0.0132 | 0 | 0 | 0 | 0 | 0 | 0 | -0.0050 |
| **PHQ1** | 0 | 0 | 0 | -0.0073 | -0.0038 | -0.0143 | 0 | 0 |
| **PHQ2** | -0.0085 | 0 | 0 | 0 | -0.0590 | 0 | -0.0695 | -0.0240 |
| **PHQ3** | 0 | 0 | 0 | 0 | -0.0006 | 0 | -0.0140 | 0 |
| **PHQ4** | 0 | 0 | -0.0149 | -0.0108 | -0.0031 | 0 | -0.0123 | 0 |
| **PHQ5** | 0 | 0 | 0 | 0 | 0 | 0 | 0 | 0 |
| **PHQ6** | -0.0153 | 0 | -0.0105 | 0 | -0.0225 | 0 | -0.0398 | 0 |
| **PHQ7** | 0 | 0 | 0 | 0 | -0.0136 | 0 | -0.0021 | 0 |
| **PHQ8** | 0 | 0 | 0 | 0 | 0 | 0 | 0 | 0 |
| **O1** | - | 0.0784 | 0.2186 | 0.0889 | 0.0253 | 0.0297 | 0.0499 | 0.0620 |
| **O2** | 0.0784 | - | 0.1602 | 0.1374 | 0 | 0 | 0.0383 | 0 |
| **O3** | 0.2186 | 0.1602 | - | 0.2508 | 0.0225 | 0 | 0.1214 | 0.0748 |
| **O4** | 0.0889 | 0.1374 | 0.2508 | - | 0.0202 | 0 | 0.1128 | 0.0186 |
| **H1** | 0.0253 | 0 | 0.0225 | 0.0202 | - | 0.2708 | 0.1123 | 0.3186 |
| **H2** | 0.0297 | 0 | 0 | 0 | 0.2708 | - | 0.1119 | 0.1905 |
| **H3** | 0.0499 | 0.0383 | 0.1214 | 0.1128 | 0.1123 | 0.1119 | - | 0.1695 |
| **H4** | 0.0620 | 0 | 0.0748 | 0.0186 | 0.3186 | 0.1905 | 0.1695 | - |
| **PCS1** | 0.0140 | 0.0180 | 0.0221 | 0.0866 | 0 | 0 | 0.0091 | 0 |
| **PCS3** | 0 | 0 | 0 | 0.0128 | 0 | 0 | 0 | 0 |
| **PCS4** | 0.0234 | 0 | 0.0038 | 0.0043 | 0 | 0 | 0 | 0 |
| **PCS6** | 0.0454 | 0 | 0.0100 | 0 | 0.0060 | 0 | 0 | 0 |
| **PCS7** | 0 | 0 | 0 | 0 | 0 | 0 | 0 | 0 |
| **PCS8** | 0 | 0 | 0.0131 | 0.0038 | 0 | 0 | 0.0234 | 0 |
| **MSSS1** | 0 | 0.0215 | 0 | 0 | 0.0121 | 0 | 0.0316 | 0.0220 |
| **MSSS2** | 0 | 0 | 0 | 0 | 0.0217 | 0 | 0 | 0 |
| **MSSS3** | 0 | 0 | 0 | 0 | 0 | 0 | 0 | 0 |
| **MSSS4** | 0 | 0 | 0 | 0 | 0 | 0 | 0.0226 | 0 |
| **MSSS7** | 0 | 0 | 0 | 0 | 0 | 0.0029 | 0 | 0.0166 |
| **MSSS8** | 0 | 0.0255 | 0.0107 | 0.0048 | 0 | 0 | 0.0160 | 0 |
| **MSSS9** | 0 | 0 | 0 | 0 | 0.0204 | 0.0268 | 0 | 0.0025 |
| **MSSS10** | 0 | 0 | 0 | 0 | 0.0010 | 0.0395 | 0.0019 | 0 |
| **MSSS11** | 0 | 0 | 0 | 0 | 0.0141 | 0 | 0 | 0.0005 |
| **MSSS12** | 0 | 0 | 0 | 0 | 0.0156 | 0.0481 | 0 | 0.0047 |
| **G1** | 0.0235 | 0 | 0.0187 | 0.0298 | 0.0072 | 0 | 0.0531 | 0.0220 |
| **G2** | 0.0076 | 0 | 0.0240 | 0.0019 | 0.0041 | 0.0008 | 0.0070 | 0 |
| **G3** | 0 | 0 | 0 | 0.0562 | 0 | 0.0005 | 0.0054 | 0.0181 |
| **G4** | -0.0235 | 0 | 0 | -0.0276 | -0.0005 | 0 | -0.0080 | -0.0140 |
| **G5** | -0.0072 | -0.0073 | 0 | 0 | -0.0137 | 0 | 0 | 0 |

*Table S3. Edge-weights for EPOCH optimism and happiness.*

|  | **PCS1** | **PCS3** | **PCS4** | **PCS6** | **PCS7** | **PCS8** |
| --- | --- | --- | --- | --- | --- | --- |
| **GAD1** | 0 | 0 | 0 | 0 | 0 | 0 |
| **GAD2** | 0 | 0 | 0 | 0 | 0 | 0 |
| **GAD4** | 0 | 0 | -0.0071 | -0.0123 | 0 | 0 |
| **GAD5** | 0 | 0 | 0 | 0 | 0 | 0 |
| **GAD6** | -0.0047 | 0 | 0 | 0 | 0 | 0 |
| **GAD7** | 0 | 0 | 0 | -0.0130 | 0 | 0 |
| **PHQ1** | 0 | 0 | 0 | -0.0220 | 0 | -0.0247 |
| **PHQ2** | 0 | 0 | -0.0445 | -0.0128 | 0 | 0 |
| **PHQ3** | 0 | -0.0207 | 0 | 0 | 0 | 0 |
| **PHQ4** | -0.0193 | -0.0046 | 0 | 0 | -0.0239 | 0 |
| **PHQ5** | 0 | 0 | 0 | 0 | 0 | 0 |
| **PHQ6** | -0.0553 | 0 | 0 | -0.0168 | 0 | 0 |
| **PHQ7** | -0.0047 | 0 | 0 | -0.0049 | 0 | 0 |
| **PHQ8** | 0 | 0 | -0.0062 | -0.0063 | -0.0097 | 0 |
| **O1** | 0.0140 | 0 | 0.0234 | 0.0454 | 0 | 0 |
| **O2** | 0.0180 | 0 | 0 | 0 | 0 | 0 |
| **O3** | 0.0221 | 0 | 0.0038 | 0.0100 | 0 | 0.0131 |
| **O4** | 0.0866 | 0.0128 | 0.0043 | 0 | 0 | 0.0038 |
| **H1** | 0 | 0 | 0 | 0.0060 | 0 | 0 |
| **H2** | 0 | 0 | 0 | 0 | 0 | 0 |
| **H3** | 0.0091 | 0 | 0 | 0 | 0 | 0.0234 |
| **H4** | 0 | 0 | 0 | 0 | 0 | 0 |
| **PCS1** | - | 0.2293 | 0.0450 | 0.1090 | 0 | 0.1839 |
| **PCS3** | 0.2293 | - | 0 | 0.0008 | 0.2025 | 0.0806 |
| **PCS4** | 0.0450 | 0 | - | 0.2274 | 0.1742 | 0.0941 |
| **PCS6** | 0.1090 | 0.0008 | 0.2274 | - | 0.4437 | 0.0280 |
| **PCS7** | 0 | 0.2025 | 0.1742 | 0.4437 | - | 0 |
| **PCS8** | 0.1839 | 0.0806 | 0.0941 | 0.0280 | 0 | - |
| **MSSS1** | 0 | 0 | 0 | 0 | 0 | 0 |
| **MSSS2** | 0 | 0.0038 | 0 | 0 | 0 | 0 |
| **MSSS3** | 0.0046 | 0.0230 | 0.0429 | 0 | 0 | 0 |
| **MSSS4** | 0 | 0 | 0 | 0 | 0 | 0 |
| **MSSS7** | 0 | 0 | 0 | 0 | 0 | 0 |
| **MSSS8** | 0 | 0 | 0 | 0 | 0 | 0 |
| **MSSS9** | 0 | 0 | 0 | 0 | 0 | 0 |
| **MSSS10** | 0 | 0 | 0 | 0 | 0 | 0 |
| **MSSS11** | 0 | 0 | 0 | 0.0382 | 0 | 0.0037 |
| **MSSS12** | 0.0030 | 0.0211 | 0 | 0 | 0 | 0 |
| **G1** | 0 | 0.0345 | 0.0062 | 0.0444 | 0 | 0 |
| **G2** | 0 | 0.0296 | 0.0389 | 0.0090 | 0.0287 | 0 |
| **G3** | 0 | 0.0780 | 0.0085 | 0 | 0 | 0 |
| **G4** | 0 | 0 | 0.0285 | 0 | 0 | 0 |
| **G5** | 0 | 0.0088 | 0 | 0.0143 | 0.0180 | 0 |

*Table S4. Edge-weights for PCS.*

|  | **MSSS1** | **MSSS2** | **MSSS3** | **MSSS4** | **MSSS7** | **MSSS8** | **MSSS9** | **MSSS10** | **MSSS11** | **MSSS12** |
| --- | --- | --- | --- | --- | --- | --- | --- | --- | --- | --- |
| **GAD1** | 0 | 0 | 0 | -0.007 | 0 | -0.002 | 0 | 0 | 0 | 0 |
| **GAD2** | 0 | 0 | 0 | 0 | -0.023 | -0.005 | 0 | 0 | 0 | -0.020 |
| **GAD4** | 0 | 0 | -0.004 | -0.003 | 0 | 0 | 0 | 0 | 0 | 0 |
| **GAD5** | 0 | 0 | 0 | 0 | 0 | 0 | 0 | 0 | -0.001 | 0 |
| **GAD6** | 0 | 0 | 0 | 0 | -0.002 | -0.016 | 0 | 0 | 0 | -0.032 |
| **GAD7** | 0 | 0 | 0 | 0 | 0 | -0.013 | 0 | 0 | -0.013 | 0 |
| **PHQ1** | 0 | 0 | 0 | 0 | -0.004 | 0 | 0 | 0 | 0 | 0 |
| **PHQ2** | 0 | 0 | 0 | 0 | 0 | 0 | 0 | 0 | -0.015 | -0.005 |
| **PHQ3** | 0 | 0 | 0 | -0.008 | 0 | -0.023 | 0 | 0 | -0.002 | 0 |
| **PHQ4** | 0 | 0 | 0 | 0 | 0 | 0 | 0 | 0 | 0 | 0 |
| **PHQ5** | 0 | 0 | 0 | -0.022 | 0 | -0.031 | 0 | 0 | 0 | 0 |
| **PHQ6** | 0 | 0 | 0 | -0.008 | -0.009 | -0.039 | 0 | 0 | 0 | -0.014 |
| **PHQ7** | 0 | 0 | 0 | 0 | 0 | 0 | 0 | 0 | 0 | -0.006 |
| **PHQ8** | 0 | 0 | 0 | -0.021 | 0 | 0 | 0 | 0 | -0.018 | 0 |
| **O1** | 0 | 0 | 0 | 0 | 0 | 0 | 0 | 0 | 0 | 0 |
| **O2** | 0.022 | 0 | 0 | 0 | 0 | 0.026 | 0 | 0 | 0 | 0 |
| **O3** | 0 | 0 | 0 | 0 | 0 | 0.011 | 0 | 0 | 0 | 0 |
| **O4** | 0 | 0 | 0 | 0 | 0 | 0.005 | 0 | 0 | 0 | 0 |
| **H1** | 0.012 | 0.022 | 0 | 0 | 0 | 0 | 0.020 | 0.001 | 0.014 | 0.016 |
| **H2** | 0 | 0 | 0 | 0 | 0.003 | 0 | 0.027 | 0.040 | 0 | 0.048 |
| **H3** | 0.032 | 0 | 0 | 0.023 | 0 | 0.016 | 0 | 0.002 | 0 | 0 |
| **H4** | 0.022 | 0 | 0 | 0 | 0.017 | 0 | 0.003 | 0 | 0.001 | 0.005 |
| **PCS1** | 0 | 0 | 0.005 | 0 | 0 | 0 | 0 | 0 | 0 | 0.003 |
| **PCS3** | 0 | 0.004 | 0.023 | 0 | 0 | 0 | 0 | 0 | 0 | 0.021 |
| **PCS4** | 0 | 0 | 0.043 | 0 | 0 | 0 | 0 | 0 | 0 | 0 |
| **PCS6** | 0 | 0 | 0 | 0 | 0 | 0 | 0 | 0 | 0.038 | 0 |
| **PCS7** | 0 | 0 | 0 | 0 | 0 | 0 | 0 | 0 | 0 | 0 |
| **PCS8** | 0 | 0 | 0 | 0 | 0 | 0 | 0 | 0 | 0.004 | 0 |
| **MSSS1** | - | 0.305 | 0.048 | 0.067 | 0.091 | 0.012 | 0 | 0.140 | 0 | 0.021 |
| **MSSS2** | 0.305 | - | 0.090 | 0 | 0.010 | 0.088 | 0.187 | 0.168 | 0 | 0.061 |
| **MSSS3** | 0.048 | 0.090 | - | 0.352 | 0 | 0.070 | 0.008 | 0.015 | 0.180 | 0 |
| **MSSS4** | 0.067 | 0 | 0.352 | - | 0.041 | 0.308 | 0 | 0.094 | 0.181 | 0 |
| **MSSS7** | 0.091 | 0.010 | 0 | 0.041 | - | 0.017 | 0.274 | 0.027 | 0 | 0.219 |
| **MSSS8** | 0.012 | 0.088 | 0.070 | 0.308 | 0.017 | - | 0.030 | 0.066 | 0.134 | 0.056 |
| **MSSS9** | 0 | 0.187 | 0.008 | 0 | 0.274 | 0.030 | - | 0.109 | 0 | 0.310 |
| **MSSS10** | 0.140 | 0.168 | 0.015 | 0.094 | 0.027 | 0.066 | 0.109 | - | 0.075 | 0 |
| **MSSS11** | 0 | 0 | 0.180 | 0.181 | 0 | 0.134 | 0 | 0.075 | - | 0.020 |
| **MSSS12** | 0.021 | 0.061 | 0 | 0 | 0.219 | 0.056 | 0.310 | 0 | 0.020 | - |
| **G1** | 0 | 0.029 | 0.032 | 0 | 0 | 0 | 0.003 | 0 | 0.033 | 0.004 |
| **G2** | 0 | 0 | 0.015 | 0.028 | 0 | 0 | 0 | 0 | 0.001 | 0.048 |
| **G3** | 0.027 | 0 | 0 | 0 | 0.008 | 0 | 0.051 | 0.026 | 0.033 | 0.003 |
| **G4** | 0.005 | 0 | 0.007 | 0.020 | 0.018 | 0 | 0.003 | 0 | 0.036 | 0 |
| **G5** | 0.007 | 0.004 | 0 | 0.032 | 0.035 | 0.021 | 0.005 | 0.046 | 0.011 | 0.048 |

*Table S5. Edge-weights for MSSS.*

|  | **G1** | **G2** | **G3** | **G4** | **G5** |
| --- | --- | --- | --- | --- | --- |
| **GAD1** | 0 | 0 | 0 | -0.0024 | 0 |
| **GAD2** | 0 | 0 | 0 | -0.0022 | -0.0110 |
| **GAD4** | 0 | 0 | 0 | 0 | 0 |
| **GAD5** | 0 | 0 | 0 | -0.0191 | 0 |
| **GAD6** | 0 | 0 | -0.0052 | -0.0346 | -0.0233 |
| **GAD7** | 0 | 0 | 0 | 0 | 0 |
| **PHQ1** | 0 | -0.0260 | -0.0036 | -0.0235 | -0.0072 |
| **PHQ2** | -0.0239 | 0 | 0 | 0 | -0.0073 |
| **PHQ3** | 0 | 0 | 0 | 0 | 0 |
| **PHQ4** | 0 | 0 | -0.0064 | -0.0276 | 0 |
| **PHQ5** | 0 | -0.0094 | 0 | -0.0005 | -0.0137 |
| **PHQ6** | 0 | 0 | 0 | 0 | 0 |
| **PHQ7** | -0.0263 | -0.0098 | 0 | -0.0080 | 0 |
| **PHQ8** | 0 | 0 | -0.0123 | -0.0140 | 0 |
| **O1** | 0.0235 | 0.0076 | 0 | 0.0464 | 0.0045 |
| **O2** | 0 | 0 | 0 | 0 | 0 |
| **O3** | 0.0187 | 0.0240 | 0 | 0 | 0.0143 |
| **O4** | 0.0298 | 0.0019 | 0.0562 | 0.0105 | 0.0445 |
| **H1** | 0.0072 | 0.0041 | 0 | 0.0553 | 0 |
| **H2** | 0 | 0.0008 | 0.0005 | 0 | 0 |
| **H3** | 0.0531 | 0.0070 | 0.0054 | 0 | 0.0149 |
| **H4** | 0.0220 | 0 | 0.0181 | 0.0285 | 0.0103 |
| **PCS1** | 0 | 0 | 0 | 0 | 0 |
| **PCS3** | 0.0345 | 0.0296 | 0.0780 | 0 | 0.0088 |
| **PCS4** | 0.0062 | 0.0389 | 0.0085 | 0.0285 | 0 |
| **PCS6** | 0.0444 | 0.0090 | 0 | 0 | 0.0143 |
| **PCS7** | 0 | 0.0287 | 0 | 0 | 0.0180 |
| **PCS8** | 0 | 0 | 0 | 0 | 0 |
| **MSSS1** | 0 | 0 | 0.0272 | 0.0048 | 0.0065 |
| **MSSS2** | 0.0289 | 0 | 0 | 0 | 0.0035 |
| **MSSS3** | 0.0321 | 0.0147 | 0 | 0.0074 | 0 |
| **MSSS4** | 0 | 0.0275 | 0 | 0.0203 | 0.0315 |
| **MSSS7** | 0 | 0 | 0.0075 | 0.0181 | 0.0345 |
| **MSSS8** | 0 | 0 | 0 | 0 | 0.0206 |
| **MSSS9** | 0.0026 | 0 | 0.0512 | 0.0028 | 0.0054 |
| **MSSS10** | 0 | 0 | 0.0261 | 0 | 0.0459 |
| **MSSS11** | 0.0325 | 0.0006 | 0.0325 | 0.0358 | 0.0108 |
| **MSSS12** | 0.0035 | 0.0475 | 0.0029 | 0 | 0.0481 |
| **G1** | - | 0.2113 | 0.1964 | 0.0178 | 0.1963 |
| **G2** | 0.2113 | - | 0.0859 | 0.2334 | 0.0536 |
| **G3** | 0.1964 | 0.0859 | - | 0.1433 | 0.1384 |
| **G4** | 0.0178 | 0.2334 | 0.1433 | - | 0.0413 |
| **G5** | 0.1963 | 0.0536 | 0.1384 | 0.0413 | - |

*Table S6. Edge-weights for GQ.*
